# Marine environmental risks from armed conflict in the Persian Gulf: Past warnings, present urgency

**DOI:** 10.1007/s13280-026-02419-6

**Published:** 2026-05-06

**Authors:** Kaveh Samimi-Namin, John A. Burt, Friedhelm Krupp, Bernhard Riegl

**Affiliations:** 1 Marine Evolution and Ecology Group, Naturalis Biodiversity Center, 2300 RA Leiden, The Netherlands; 2 Department of Biology, University of Oxford, Oxford, OX1 3SZ UK; 3 Natural History Museum, Cromwell Road, London, SW7 5BD UK; 4 Mubadala Arabian Center for Climate and Environmental Sciences, New York University Abu Dhabi, Abu Dhabi, United Arab Emirates; 5 Beacon Development, King Abdullah University of Science and Technology, Thuwal, Saudi Arabia; 6 Senckenberg Research Institute and Museum of Nature, Senckenberganlage 25, 60325 Frankfurt a.M., Germany; 7 Halmos College of Arts and Sciences, National Coral Reef Institute, Nova Southeastern University, Dania Beach, FL 33004 USA

**Keywords:** Coastal infrastructure, Desalination, Ecological vulnerability, Environmental preparedness, Transboundary risk

## Abstract

The Persian Gulf is both an ecologically fragile marine system and a global energy chokepoint. Past conflicts have shown that warfare in the region can cause extensive and persistent damage to coastal and marine habitats. Today, that risk is amplified by the Gulf’s shallow, semi-enclosed character, restricted exchange with the open ocean, extreme temperature and salinity, expanding hypoxia, and heavy reliance on desalination and other seawater-dependent infrastructure. Armed conflict could therefore trigger oil and chemical releases, chronic contamination, and habitat degradation, with cascading consequences for fisheries, water security, shipping, and industrial operations. Because these impacts are foreseeable and could be severe, prolonged, and transboundary, environmental preparedness in the Gulf should be integrated into regional security, infrastructure protection, and emergency planning.

## A vulnerable marine ecosystem at a global energy chokepoint

The Persian Gulf harbours one of the world’s most extreme and geopolitically sensitive marine environments. Past conflicts in this region have shown that military escalation can cause not only severe human and cultural losses, but also long-lasting environmental damage (Price et al. 1994; Krupp et al. 1996), yet the lessons of those conflicts remain largely unheeded. Today, that danger is amplified by the Gulf’s ecological fragility and by the high concentration of oil and gas facilities and other seawater-dependent infrastructure along its shores. In this shallow, semi-enclosed basin, armed conflict could trigger one of the most serious marine environmental crises of the modern era. We argue that these risks are sufficiently foreseeable that environmental preparedness in the Gulf should be integrated into regional infrastructure protection, water security, and transboundary emergency response.

The Gulf’s vulnerability reflects the combination of extreme environmental conditions, concentrated industrial use, and restricted exchange with the open ocean. It supports distinctive biodiversity adapted to naturally harsh conditions, including endemic species, although biodiversity across much of the basin remains incompletely documented (Sheppard et al. 2010; Riegl and Purkis 2012; Samimi-Namin and Hoeksema 2023). Exchange with the open ocean occurs through the narrow Strait of Hormuz, one of the world’s most important energy chokepoints (Sheppard et al. 2010; Sale et al. 2011). These features make the Gulf unusually vulnerable to disturbances affecting both marine ecosystems and essential coastal services. At the same time, coastal development, intensive desalination, widespread land reclamation, dredging, and resource extraction already place heavy pressure on marine habitats, including a mosaic of highly productive and diverse coastal ecosystems (Sheppard et al. 2010; Sale et al. 2011; Burt et al. 2020).

The Gulf’s unusual oceanography and climate intensify its susceptibility to conflict-related disturbance. It is a shallow, semi-enclosed sea with strong temperature and salinity extremes,long turnover times, and restricted exchange with the open ocean (Sheppard et al. 2010; Alosairi et al. 2011; Riegl and Purkis 2012). Summer sea-surface temperatures regularly exceed the tolerance limits of many tropical marine organisms (> 36 °C); salinity is among the highest recorded in the world’s seas because of intense evaporation and minimal freshwater input (> 44 PSU in open water and > 55 in embayments), and recurrent summer hypoxia affects parts of the basin and has intensified in recent decades (Sheppard et al. 2010; Lachkar et al. 2022). Organisms that persist under these conditions may have limited capacity to absorb additional acute stress (Burt et al. 2020), potentially increasing their vulnerability to conflict-related contamination.

Despite these extreme environmental conditions, the Gulf harbours ecologically and economically important habitats, including coral reefs, seagrass beds, mangrove stands, salt marshes, mudflats, and extensive soft-sediment communities (Sheppard et al. 2010; Sale et al. 2011; Riegl and Purkis 2012). These habitats stabilise shorelines, maintain biodiversity, and provide nursery grounds for fish and invertebrates, thereby sustaining fisheries of great economic importance (Sheppard et al. 2010; Sale et al. 2011; Riegl and Purkis 2012). Gulf coral assemblages also provide a natural model for understanding persistence under climatic extremes because of their exceptional heat tolerance (Riegl and Purkis 2012; Burt et al. 2020).

Restricted circulation further heightens vulnerability. With the Strait of Hormuz as the Gulf’s only connection to larger and better-buffered water masses, pollutants introduced into Gulf waters may accumulate in sediments and biota rather than disperse rapidly, increasing the likelihood of long-lasting contamination (Alosairi et al. 2011; Lachkar et al. 2022). Oil, heavy metals, and other contaminants may therefore exert chronic rather than transient effects. Recovery may in turn be constrained by harsh background conditions, impaired local recruitment, and limited replenishment from outside the basin. In this setting, acute damage can more easily become chronic degradation, and additional disturbance may push some habitats beyond recovery thresholds.

## Conflict could trigger multiple cascading marine hazards

Armed conflict affects biodiversity and ecosystems through direct and indirect pathways, including habitat degradation, pollution, and damage to critical coastal and offshore infrastructure that can lead to secondary contamination and broader environmental disruption (Lawrence et al. 2015). In the Persian Gulf, these risks are amplified by the dense concentration of energy infrastructure close to shore. Direct strikes, secondary accidents, or operational failures could therefore release large quantities of oil and chemicals into coastal and marine environments.

Past conflicts in the Gulf illustrate this risk clearly. During the Iran–Iraq War (1980–88), tanker attacks and associated oil spills exposed the susceptibility of Gulf coastal environments to wartime disturbance. The 1991 Gulf War then provided the clearest evidence of the scale and persistence of ecological damage that war can produce (Price et al. 1994). An estimated 7 million barrels of crude oil were intentionally released into the Gulf, and up to 1.9 million barrels of hydrocarbons contaminated intertidal habitats along about 700 km of coastline from Kuwait into Saudi Arabia, reaching as far as Qatar (Krupp et al. 1996; Jones et al. 2008). Fisheries and shallow coastal ecosystems were affected, but the most severe and persistent impacts were concentrated in intertidal habitats, especially mudflats, salt marshes, mangroves, and other coastal wetlands (Price et al. 1994; Jones et al. 1998).

Damage to intertidal habitats such as mangroves, mudflats, salt marshes, and sabkha margins reduces nursery function for fishes and invertebrates, with cascading consequences for fisheries and food webs (Price et al. 1994; Jones et al. 2008; Sale et al. 2011). These impacts reflect the multiple pathways through which oil damages marine systems, including toxicity, smothering of coastal vegetation, and chronic sediment contamination affecting benthic food webs and higher trophic levels (Price et al. 1994; Barron et al. 2020).

Consistent with these multiple pathways of damage, post-war assessments varied across marine habitats. Initial studies conducted between 1991 and 1995 suggested recovery times similar to those reported for major oil spills elsewhere in the world (Krupp et al. 1996; Jones et al. 1998). Later surveys, however, revealed a more persistent pattern of damage, including large volumes of oil buried beneath sand and extensive tar mats that remained for decades (Jones et al. 2008). Intertidal habitats were affected most severely: mangroves, salt marshes, tidal flats, sand beaches, and rocky shores showed markedly lower species diversity than unimpacted sites, and recovery was expected to be slowest in low-energy habitats without tidal flushing, particularly salt marshes, where it was projected to take more than a century (Jones et al. 2008). By contrast, some studies suggested a more mixed picture in certain subtidal habitats, where some seagrass beds and coral reefs showed no clear evidence of long-term degradation (Kenworthy et al. 1993; Vogt 1995).

These studies provide important lessons, but their implications should not be overstated. Because many assessments were geographically limited and systematic pre-war baseline data were lacking for numerous sites, both damage assessment and estimates of recovery remained constrained. What distinguishes present and future risks from past conflicts is not only the possibility of one or more large spills, but also the likelihood of more spatially dispersed releases. Whereas past releases were relatively concentrated, the much greater expansion of coastal infrastructure now increases the likelihood of multi-source contamination across the basin.

Beyond oil, conflict could introduce hazardous chemicals, airborne contaminants, heavy metals, underwater noise, and, in extreme cases, radiological contamination from damage to nuclear facilities. Damage to petrochemical and industrial facilities could release hazardous chemicals, while combustion of petroleum products could generate airborne contaminants that later deposit into Gulf waters. Military debris and unexploded ordnance may introduce heavy metals and other toxic substances into sediments (Lawrence et al. 2015).

In the Gulf, the consequences of marine pollution do not end with ecological damage. Because essential services depend heavily on seawater, marine environmental damage can quickly become a water security and infrastructure security crisis. Oil or chemical contamination, as well as direct damage to desalination plants, intake systems, and coastal industrial infrastructure, could disrupt drinking water production and shut down cooling water systems in power stations and other industries. Such disruption could also release treatment chemicals or other contaminated process waters into already stressed coastal environments (Roberts et al. 2010; Paparella et al. 2022).

The consequences of such damage would not necessarily remain confined to the Gulf. Water masses exiting through the Strait of Hormuz influence the Gulf of Oman and northern Arabian Sea, creating pathways for pollutant transport beyond the immediate disturbance zone (Alosairi et al. 2011). Environmental damage would therefore become a transboundary problem, affecting downstream habitats, migratory corridors, fisheries, and coastal economies across a wider region.

## Preparedness must catch up with foreseeable risk

In the Gulf, marine pollution can cascade directly into essential services, critical infrastructure, and broader security concerns. The central policy issue is therefore not whether armed conflict could damage the Gulf’s marine environment, but whether the region’s planning and response capacity remain adequate for damage that is both foreseeable and potentially severe. Previous conflicts showed that environmental damage can be extensive, transboundary, long-lasting, and costly to remediate; yet baseline ecological data, shared monitoring systems, and coordinated response capacity remain inadequate across the region (Price et al. 1994; Gower et al. 2025). Many of the measures needed to reduce these risks were identified more than a decade ago (Sheppard et al. 2010; Sale et al. 2011), but they have not been implemented at the level now required and, in some cases, have been ignored altogether. Those recommendations therefore remain urgent, because the Gulf’s underlying ecological and infrastructural vulnerabilities will persist even if immediate tensions subside. Existing regional mechanisms provide an important institutional starting point, but they have not yet produced the sustained operational preparedness that current risks require. The region therefore needs a more durable and operational framework for monitoring, emergency coordination, and environmental assessment—one that can remain functional even during periods of severe political tension.

Any such framework must also reflect the realities of conflict. Full regional coordination, extensive field operations, and cross-border scientific collaboration may be difficult or impossible during active conflict. The priorities outlined here should therefore be understood across three phases: first, as measures established in advance during peacetime or periods of heightened tension; second, as measures maintained in reduced or emergency form during active conflict where safety and access permit; and third, as measures for rapid assessment and remediation in the immediate post-conflict period. Framed in this way, the goal is not to assume ideal cooperation under wartime conditions, but to reduce foreseeable damage through advance preparation, limited emergency capacity, and rapid post-conflict response.

Three priorities follow directly from these lessons. First, Gulf countries and international partners should strengthen coordinated monitoring networks capable of rapidly detecting pollution events, habitat damage, and ecological change across both intertidal and subtidal systems. A central component of this effort must be the urgent consolidation and centralisation of biodiversity baselines, since the lack of systematic ecological data before 1991 hindered post-war damage assessment and weakened remediation efforts (Price et al. 1994; Sheppard et al. 2010). Existing survey data should therefore be standardised, archived in accessible repositories, and expanded through targeted assessments of ecosystems and locations most exposed to conflict-related risk. During active conflict, such systems may operate only in reduced form and depend more heavily on remote sensing, automated observations, sentinel sites, and rapid reporting from accessible areas. Even partial continuity, however, would improve damage assessment and early response.

Second, contingency planning for accidental oil and chemical releases should be updated to explicitly prioritise sensitive habitats and critical coastal infrastructure. Response plans should identify where exposure is most likely to cause long-lived ecological damage, where desalination and industrial cooling systems are most vulnerable, and where multi-hazard events could exceed local response capacity. Scientific institutions should support this effort by identifying vulnerable ecosystems, improving circulation and pollutant dispersal models, and evaluating recovery potential under different disturbance scenarios. During active conflict, response may be constrained by insecurity, damaged logistics, or restricted access, but pre-agreed priorities and protection protocols could still help reduce the most severe impacts where intervention remains possible.

Third, environmental risk assessments for ports, tanker routes, offshore energy infrastructure, desalination plants, and coastal industrial zones should explicitly account for biodiversity impacts, transboundary exposure pathways, and the need for cross-border coordination. Much of this work must occur before conflict, when access to infrastructure, data, and planning institutions is still available. After conflict, the same assessments would help prioritise remediation, restore critical services, and identify areas where environmental damage may continue to spread across borders. In the Gulf, environmental damage should not be treated as a secondary or downstream issue. It is directly linked to water security, industrial operations, and regional resilience.

Past environmental disasters have repeatedly shown that early intervention reduces long-term ecological and economic losses, whereas delayed response often allows damage to become entrenched. In the Persian Gulf, that lesson is especially important, because a major conflict would likely leave an environmental legacy lasting decades. Recognising these risks does not require predicting how any specific crisis will unfold; it requires acknowledging that the environmental consequences of warfare in this region are predictable and can be reduced through advance planning.

The Persian Gulf is one of the clearest examples of a marine system in which fragile ecosystems and strategic infrastructure are tightly interwoven. Environmental protection here is not separate from regional security planning; it is part of it.
